# Tuning the catalytic acidity in Al_2_O_3_ nanofibers with mordenite nanocrystals for dehydration reactions†

**DOI:** 10.1039/d2cy00143h

**Published:** 2022-05-11

**Authors:** M. A. Rodriguez-Olguin, R. N. Cruz-Herbert, H. Atia, M. Bosco, E. L. Fornero, R. Eckelt, D. A. De Haro Del Río, A. Aguirre, J. G. E. Gardeniers, A. Susarrey-Arce

**Affiliations:** Mesoscale Chemical Systems, MESA+ Institute, University of Twente PO. Box 217, 7500AE Enschede The Netherlands j.g.e.gardeniers@utwente.nl a.susarreyarce@utwente.nl; Universidad Autónoma de Nuevo León, Facultad de Ciencias Químicas Pedro de Alba S/N San Nicolás de los Garza Nuevo León 64455 Mexico david.dharodlr@uanl.edu.mx; Leibniz Institute for Catalysis Albert-Einstein-Straße 29a D-18059 Rostock Germany; Instituto de Desarrollo Tecnológico para la Industria Química (INTEC), Universidad Nacional del Litoral CONICET, Güemes 3450 S3000GLN Santa Fe Argentina aaguirre@santafe-conicet.gov.ar; Facultad de Ingeniería Química, Universidad Nacional del Litoral (UNL) Santiago del Estero 2829 Santa Fe 3000 Argentina; Facultad de Ingeniería en Ciencias Hídricas, UNL, Ciudad Universitaria Ruta Nacional N° 168 – Km 472,4 3000 Santa Fe Argentina

## Abstract

Alumina (Al_2_O_3_) is one of the most used supports in the chemical industry due to its exceptional thermal stability, surface area, and acidic properties. Mesoscopic structured alumina with adequate acidic properties is important in catalysis to enhance the selectivity and conversion of certain reactions and processes. This study introduces a synthetic method based on electrospinning to produce Al_2_O_3_ nanofibers (ANFs) with zeolite mordenite (MOR) nanocrystals (hereafter, hybrid ANFs) to tune the textural and surface acidity properties. The hybrid ANFs with electrospinning form a non-woven network with macropores. ANF-HMOR, *i.e.*, ANFs containing protonated mordenite (HMOR), shows the highest total acidity of *ca.* 276 μmol g^−1^ as determined with infrared spectroscopy using pyridine as a molecular probe (IR-Py). IR-Py results reveal that Lewis acid sites are prominently present in the hybrid ANFs. Brønsted acid sites are also observed in the hybrid ANFs and are associated with the HMOR presence. The functionality of hybrid ANFs is evaluated during methanol dehydration to dimethyl ether (DME). The proof of concept reaction reveals that ANF-HMOR is the more active and selective catalyst with 87% conversion and nearly 100% selectivity to DME at 573 K. The results demonstrate that the textural properties and the acid site type and content can be modulated in hybrid ANF structures, synergistically improving the selectivity and conversion during the methanol dehydration reaction. From a broader perspective, our results promote the utilization of hybrid structural materials as a means to tune chemical reactions selectively.

## Abstract 2

The chemical and structural properties of Al_2_O_3_ are tuned for dehydration reactions. The synergy between the structured Al_2_O_3_ shaped as nanofiber and the acid site nature of the zeolite mordenite in the nanofiber improves the dehydration reaction.

## Graphical abstract

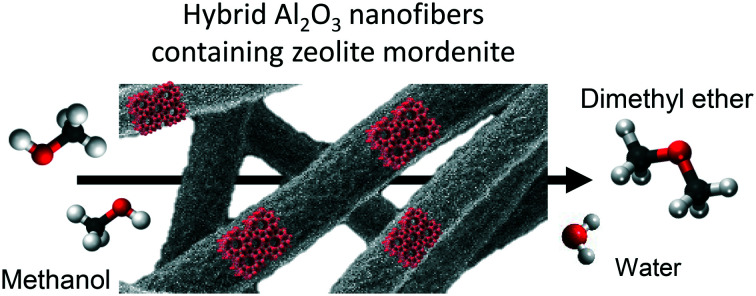

## Introduction

Al_2_O_3_ in heterogeneous catalysis has been used to support alkali metals, noble metals, and metal oxides with a wide range of applications in biomass upgrading, the oil industry, and the automotive sector.^1–8^ Such a wide range of applications is due to the various Al_2_O_3_ polymorphs,^9,10^ each with unique properties, such as thermal stability, acidity, and high specific surface area, targeted for specific reactions.^11–16^ Besides the current synthetic approaches in Al_2_O_3_,^16–24^ structuring of mesoscopic scale Al_2_O_3_ remains a challenge. Typical methods to structure Al_2_O_3_ are extrusion,^8^ injection molding,^25^ and 3D printing.^25,26^ These methods aim to improve reaction rates *via* mass transfer and diffusivity by varying the Al_2_O_3_ geometries. Although such Al_2_O_3_ structures are often relevant in catalysis, sufficient attention should be paid to the chemical properties of Al_2_O_3_, such as the nature of the acid sites to promote acidity in structural acid catalysts.

Electrospinning is a convenient method that provides sufficient versatility to optimize chemical properties in structured materials.^27–36^ The method produces nanofiber-like structures formed during the withdrawal of a jet from a droplet subjected to an external electric field, which later is deposited over a collector plate for further treatment.^37–39^ Although the approach has been primarily used in the biomedical field,^40^ it is increasingly used for energy storage, energy conversion, and catalysis.^41–43^ In the past, ceramic materials, including alumina, have been subject to modifications that have led them to have one-dimensional (1-D) configurations, such as nanofibers.^44^ These arrangements have attracted attention due to the unique functionalities provided by nanofibers, for example, high mechanical strength, high surface-area/weight ratio, chemical composition, and stability.^44–46^ Recently, M. A. Rodriguez-Olguin *et al.*^43^ demonstrated that the acid site content can be enhanced in ANFs. The authors use various aluminum precursors during electrospinning. From the precursor assessment, aluminum di(*sec*-butoxide)acetoacetic ester chelate (ASB) is identified as an ideal precursor for obtaining ANFs with a large amount of weak and medium strength Lewis acid sites (LASs). Furthermore, ASB chelate promotes mesopores of 2–50 nm in size.^47–51^ Macropores (*i.e.*, >50 nm), especially relevant in catalysts, can also be observed between the fiber-to-fiber interspaces.^43^ From this perspective, it is fair to say that the benefit of nanofibers relies on their hierarchy having multiple levels of porosity that combine meso/macropores.

Similarly, multilevel porosity can be found in hierarchical catalysts, such as zeolites. Zeolites have pore sizes ranging from micropores to mesopores and macropores.^52–54^ These pores are composed of Si and Al atoms coordinated with oxygen, forming channel networks of diverse sizes. However, if the zeolite pores are too small, the reaction might be diffusion or mass transport limited. Synthetic methods to increase the availability of meso/macropores have been established to reduce transport issues. A widely applied approach in zeolites is leaching.^55^ However, leaching involves several synthetic steps that are composition and zeolite type dependent. An interesting alternative is providing structure to existing catalysts, such as zeolites. Zeolites are compatible with synthetic approaches, such as electrospinning.^56–62^ A key aspect of electrospinning is that it can facilitate the formation of macropores without limiting mesopore formation.^43^

Reports have demonstrated advantages for shaped zeolites as either single or hollow nanofibers.^56,57^ This includes crystalline fibers of zeolite Y,^58,59^ ZSM-5,^60,61^ and SUZ-4.^62^ Other approaches used to structure zeolites involve templates or zeolite mixtures with more materials to create composites. For example, F. Ocampo *et al.*^63^ developed a multimodal pore size distribution using a zeolite and a glass monolith. The authors demonstrate the HZSM-5/glass monolith functionality during n-hexane cracking. Following a similar concept, zeolite Y, MFI, or beta on α-SiC foams, carbon nanotubes, or TiO_2_ nanofibers have been synthesized and tested during a catalytic reaction.^64–66^ From the previous examples, MOR shaped as nanofibers or MOR composites within a nanofiber are limited but increasingly recognized as an effective way to enhance the conversion and selectivity in chemical reactions, such as CO_2_ methanation using a silica MOR composite.^67^

The rationale behind using MOR in structured materials as a composite is to find more dimensionally refined systems that allow easy access to molecules to adsorb, react and desorb over LASs and Brønsted acid sites (BASs).^68^ This is the case for acid catalysts, widely used for alcohol dehydration reactions, such as methanol dehydration to DME.^69,70^ An accepted mechanism for the mentioned reaction occurs with the adsorption of the alcohols over a LAS or BAS, and an adjacent LAS forming two species, which produce DME and water upon condensation.^71^ However, in this reaction, a tradeoff between the LAS and BAS strength has to be found because it can significantly alter methanol dehydration products.^72^ It is generally accepted that the DME synthesis preferably proceeds on a solid acid catalyst with weak and moderate acidic sites. For strong BASs commonly found in MOR,^73,74^ a considerable amount of side-products can be formed. The products consist predominantly of hydrocarbons or coke, which affect the selectivity and lifespan of the catalyst. An alternative that can compensate for high BAS contents is a composite, for example, MOR with Al_2_O_3_ shaped as nanofibers.^75^ Al_2_O_3_ (ref. 76–81) is a known catalyst used to dehydrate methanol and produce DME by following a proposed mechanism based purely on LASs.^82,83^ From this perspective, a synergy between acid catalysts containing LASs and BASs has to be found to tune chemical reactions like DME synthesis selectively.

The present work synthesized nanofibers composed of amorphous Al_2_O_3_ and MOR with electrospinning. The synergy of MOR and amorphous Al_2_O_3_ is demonstrated by comparing hybrid ANFs with Al_2_O_3_ shaped as nanofibers. The added value of the structured fibers is assessed by comparing hybrid ANFs against Al_2_O_3_ and MOR without a nanofiber shape. Structural and morphological analysis indicates the presence of MOR in the ANFs. Textural analysis corroborates our findings, where a decrease in the surface area for hybrid fibers is observed. Furthermore, hybrid ANFs show the highest acidity as determined with IR-Py. The acid sites present in the hybrid ANFs are LASs and BASs, while for the control samples of Al_2_O_3_, only LASs have been found. The functionality of hybrid ANFs is assessed during methanol dehydration to DME as a proof of concept reaction. The results reveal a synergetic effect between Al_2_O_3_ and MOR in the nanofibers and demonstrate the added value of hybrid materials in chemical reactions.

## Methodology

### Microwave-assisted zeolite synthesis

Microwave-assisted hydrothermal synthesis was carried out to produce NaMOR nanocrystals. Colloidal silica (Ludox HS-40, 40% w/w, Aldrich), Al(OH)_3_ (98%, Aldrich), and NaOH (98%, Sigma-Aldrich) were used as precursors to obtain an initial gel with a ratio of 6 NaOH : Al_2_O_3_ : 30 SiO_2_ : 780 H_2_O. In a typical run, 1.6 g of Al(OH)_3_ was dissolved in 2.2 g of NaOH (98%, Sigma-Aldrich) and 61 g of deionized water. Then, 11.4 g of colloidal silica was added until complete dissolution, and 3 g of MOR nanocrystals from Zeolyst were used as seeds to enhance the crystallization rate. The resulting suspension was stirred for 1 h at 450 rpm. After, the obtained gel was placed in a Teflon autoclave that was put in a Milestone Flexiwave microwave. The crystallization conditions followed a ramp of 20 K min^−1^ to reach 453 K and used 600 W as the maximum power for a synthesis time of 1 h. This strategy allows the reduction of the synthesis time that has traditionally been reported from 24–48 h. Finally, the material was recovered by filtration, washed to a pH lower than 9, and dried at 343 K for 24 h in air. The final Si/Al ratio as determined with EDX is 10.5 for all NaMOR nanocrystals, which are then used during the ionic exchange (see below).

### Protonation of zeolite mordenite

The protonation of the NaMOR nanocrystals is followed by post-treatment using 1 M NH_4_NO_3_ (ACS grade, Sigma Aldrich) solution in deionized water. First, NaMOR nanocrystals were dispersed in NH_4_NO_3_ solution, 1 g of solid per 10 ml of solution, and stirred at 353 K for 2 h. Then, the material was washed and filtered once the time had elapsed. The product obtained was dried at 373 K in air for 12 h, and once the time has expired, the ion exchange was repeated 3 times. At the end of the ion exchanges, the samples were calcined at 773 K for 3 h in air. The material obtained was labeled HMOR.

### Hybrid fiber synthesis

The hybrid ANFs containing either NaMOR or HMOR were prepared by electrospinning using a commercial electrospinning system from IME Technologies (The Netherlands). The IME system was operated utilizing a stainless-steel needle of 0.4 mm inner diameter at a separation distance of 12 cm from the aluminum collector plate. First, a mixture consisting of 4% p/v C_14_H_27_AlO_5_ (ASB) technical grade from Alpha Aesar, 6% p/v polyvinylpyrrolidone (PVP, MW ∼1 300 000), and 0.26% p/v *t*-octylphenoxypolyethoxyethanol (Triton ×100, Sigma-Aldrich) dissolved in ethanol (100% Tech. grade, BOOM B.V., The Netherlands) was used as the aluminum precursor solution to generate ANFs. To make the hybrid ANFs, the synthesized NaMOR or HMOR nanocrystals were incorporated into the ASB solution, reaching a final concentration of 0.33% p/v in each case. The prepared solutions were electrospun at environmental temperature and humidity using a potential of 18 kV at an 8 mL h^−1^ infusion rate. After fiber deposition, all-fiber samples were dried in an oven at 353 K for 12 h to remove the excess solvent. Subsequently, they were calcined (Nabertherm LH 15/12) in air with a temperature ramp of 0.5 K min^−1^ to 623 K for 3 h and then 1 K min^−1^ until reaching 773 K for 4 h to ensure the production of amorphous alumina.^43^ Hereafter, the obtained hybrid ANFs are named ANF-NaMOR and ANF-HMOR for simplicity. It should be noted that commercial MOR (CBV 10A, Zeolyst) was also used following the previously described hybrid nanofiber preparation. Control samples of particulate alumina (Al_2_O_3_-NP) and particulate alumina containing HMOR (Al_2_O_3_-HMOR-NP) were prepared by dropcasting using the same ASB, NaMOR, HMOR precursor solutions in crucibles. These samples were annealed following the same procedure as ANF-NaMOR and ANF-HMOR samples.

## Characterization

### Morphological characterization

High-resolution (HR)-SEM images of samples were taken using a Zeiss MERLIN SEM microscope operated at 1.40 kV coupled with a high-efficiency secondary electron detector (HE-SE2). SEM-scanning transmission electron microscopy (STEM) was performed at 20 kV. Prior to STEM analysis, samples were sonicated in ethanol, which led to the fragmentation of the fibers into smaller fiber pieces.

### Structural characterization

The crystalline structure of NaMOR and HMOR nanocrystals was analyzed with a Siemens (D5000, E04-0012 series) diffractometer, using CuKα radiation (*λ* = 1.5418 Å) operated at 35 kV, 25 mA, in the 2*θ* range between 5 and 50°, employing a step size of 0.02° min^−1^ and a step time of 4 s. The hybrid fibers were analyzed by X-ray powder diffraction (D2 PHASER, Bruker) using Cu Kα radiation (*λ* = 1.5418 Å) operated at 30 kV, 10 mA, in the 2*θ* range between 7 and 45°, employing a step size of 0.05° and a scan speed of 0.1° s^−1^. A Si low background sample holder (Bruker) was used for the hybrid samples.

### Chemical characterization

X-ray photoelectron spectroscopy (XPS) general survey analysis was performed with a Quantera SXM machine from Physical Electronics using monochromated Al Kα (1486.6 eV). All samples were fixed in a stainless-steel holder. A low energy electron flood gun was used to supply the missing photo- and Auger electrons. The electron binding energies were referenced to aliphatic carbon C 1s at 284.8 eV. The obtained peak analysis was made using the PHI Multipak V9.9.0.8 software (Physical Electronics, Inc.).

### Textural analysis

The BET surface area, pore-volume, and pore diameter of the samples were determined from the nitrogen adsorption/desorption isotherms at 77 K on a Micrometrics ASAP 2010 instrument. Before the measurement, each sample was evacuated at 473 K for 4 h. The pore size distributions were calculated from the desorption branch of the isotherm using the Barrett–Joyner–Halenda (BJH) model.^54^

### NH_3_-TPD

To determine the total acidity properties of the samples, NH_3_-TPD analysis was performed using a Micromeritics Autochem II 2910 instrument. Prior to NH_3_ adsorption, 150 mg of the sample was loaded into a U-shaped quartz reactor and heated from RT to 673 K with 10 K min^−1^ in a flow of He (50 ml min^−1^), held for 30 min at 673 K (to remove any adsorbed species on the surface). After that, the reactor was cooled to 373 K. The sample was saturated with 1% NH_3_ in He (50 ml min^−1^) for 120 min at 373 K, followed by helium flushing (50 ml min^−1^) for 60 min at 373 K to remove physisorbed NH_3_. The sample was then heated to 1073 K at a rate of 10 K min^−1^ in He flowed (50 mL min^−1^) and held at 1073 K for 30 min for NH_3_ desorption. The effluent gases were analyzed with a quadrupole mass spectrometer (Balzers Omnistar) using *m*/*z* = 15.

### IR measurements and pyridine adsorption

The nature and strength of acid sites were determined through pyridine (Py) adsorption over the materials and subsequent temperature-programmed desorption (TPD-Py). The samples were analyzed with IR spectroscopy through *in situ* transmission on self-supported wafers (10–15 mg, 13 mm in diameter) pressed at 5 t cm^−2^ (490 MPa). The wafers were placed in a Pyrex IR cell fitted with water-cooled NaCl windows. More details of the experimental setup can be found elsewhere.^84^ Before the adsorption experiments, each sample was pretreated *in situ* at 723 K (10 K min^−1^) for 30 min under a N_2_ flow (50 sccm), then cooled down to 303 K, and the reference IR spectra of the “clean wafer” were taken. The samples were exposed to a flow of N_2_ containing evaporated Py. The physisorbed Py was further removed under flowing N_2_ until the spectra of the adsorbed Py remained stable (about 60 min). The thermal desorption of Py was measured from 303 K to 723 K with 5 K min^−1^ in a flow of N_2_ (50 ml min^−1^). The spectra were acquired with a Nicolet Magna 550 FTIR spectrometer with a cryogenic MCT detector (4 cm^−1^ resolution, 25 scans). The gas used in this study was high purity grade N_2_ (INDURA UHP 99.999%) and was further purified through molecular sieves (3 Å), and MnO/Al_2_O_3_ traps to remove water and oxygen impurities, respectively.

### Catalytic test

The synthesized materials were tested for the dehydration of methanol to dimethyl ether (DME) between 423 and 723 K (heating ramp of 1 K min^−1^) in a fixed bed glass tubular microreactor (i.d. = 5.3 mm). The reactor was loaded with 50 mg of catalyst diluted (1 : 5) with milled quartz (200 mesh). The methanol concentration was 7% v/v in Ar obtained from a gas saturator filled with pure methanol immersed into a thermostatic bath. The total flow rate was set to 20 mL min^−1^, giving a weight hourly space velocity (WHSV) of 2.3 g_methanol_ g_cat_^−1^ h^−1^. The pipelines were heated to prevent methanol and product condensation. Before the catalytic test, the samples were pretreated at 673 K under a flow of Ar (50 mL min^−1^) for 1 h. The outlet gas stream was analyzed continuously with a mass spectrometer Prisma QMG220 (Pfeiffer). The following mass/charge signals were recorded: 2(H_2_), 16 (CH_4_), 18 (H_2_O), 28 (CO), 29, 31 and 32 (methanol), 40 (Ar), 44 (CO_2_), 45 (DME), and 58 and 59 (olefins).

## Results and discussion

### Hybrid ANFs containing MOR

**Fig. 1:**
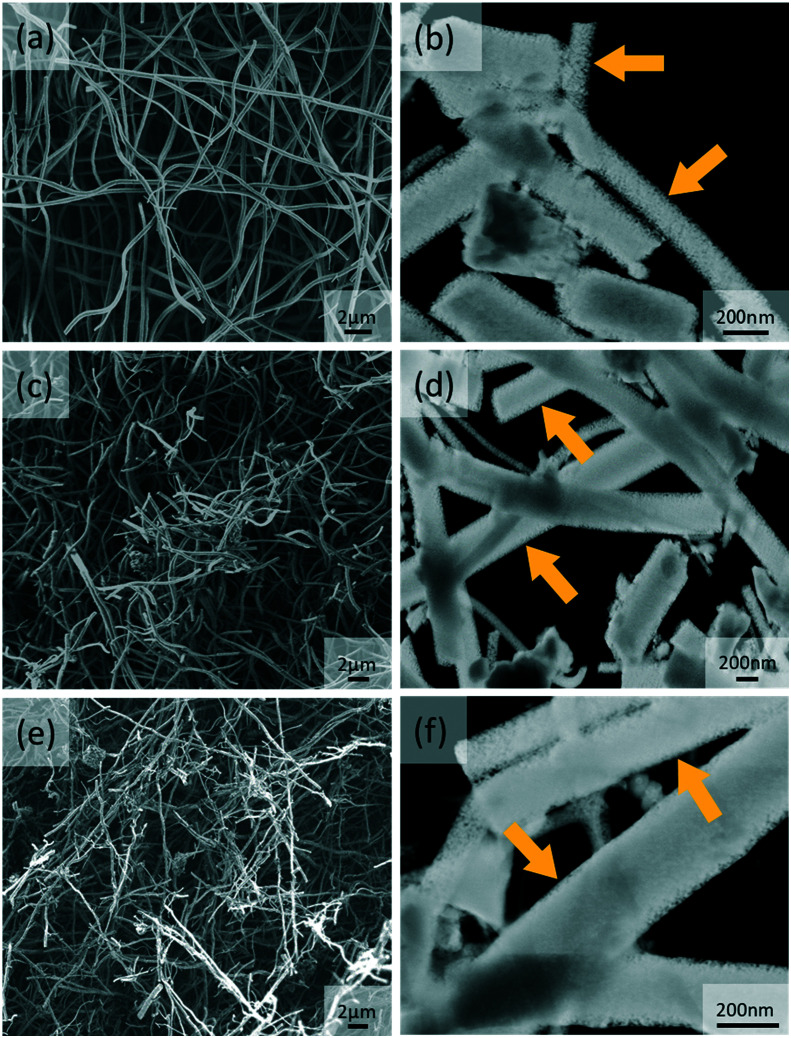
SEM and dark-field STEM images of (a and b) ANFs, (c and d) ANF-NaMOR and (e and f) ANF-HMOR. The yellow arrows highlight the pore openings in the fibers.

The synthesis of the hybrid ANFs started by selecting the MOR crystallite size. Commercial NaMOR, which has an average crystallite size of 220 nm (Fig. S1a†), has been used during electrospinning. However, it is found that this leads to severe heterogeneity after annealing (Fig. S1b†). This heterogeneity is attributed to the relatively large (compared to the nanofiber dimensions) crystallite size of NaMOR, which upon annealing, promotes nanofiber instability leading to hybrid ANFs with an irregular shape (Fig. S1b†). In contrast to the commercial NaMOR, the synthesized NaMOR shown in Fig. S2† has smaller crystallite sizes ranging between 110 and 118 nm. The crystallite size is nearly half of the nanofiber diameter. From the results, small MOR crystallites can lead to less heterogeneity in ANFs, as shown in Fig. 1. In this figure, the hybrid ANFs (Fig. 1c and e) retain their nanofiber shape, similar to ANFs in Fig. 1a that show a non-woven fiber morphology. The estimated nanofiber diameters are 321 ± 74 nm for ANFs, 315 ± 120 nm for ANFs containing NaMOR (ANF-NaMOR), and 241 ± 76 nm for ANFs containing HMOR (ANF-HMOR).

A detailed structural analysis using dark-field STEM images is also presented in Fig. 1b, d and f to provide insights into the morphology of hybrid ANFs. From the images, less dense ANF nanofibers are found in Fig. 1b. The effect becomes evident at the edges of the ANFs, with small pore openings (see yellow arrows). In contrast, STEM images of ANF-NaMOR and ANF-HMOR in Fig. 1d and f show denser areas at the borders and center of the fiber structure, possibly due to MOR nanocrystals inside the fibers, which could act as a ‘filler’ material densifying the nanofibers. It should be noted that the high amount of broken fibers is due to the sonication used during specimen preparation for STEM. To this end, EDS mapping in Fig. S3† is used to verify the hybrid nanofiber constitution and generate insights into the MOR distribution by looking at the silicon Kα1 signal. The silicon signal has been found in the non-woven structure and specific densified areas.

**Fig. 2:**
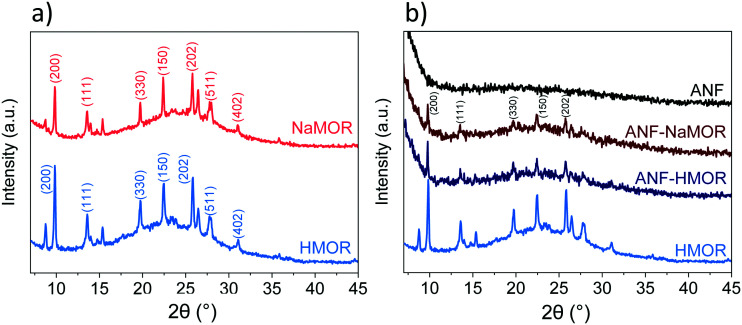
XRD patterns of a) bare NaMOR and HMOR nanocrystals and b) ANFs, ANF-NaMOR, and ANF-HMOR.

The chemical composition at the surface of the hybrid ANFs from Fig. 1 is analyzed with XPS to determine the types of species present on the hybrid ANFs (Fig. S4 and Table S1†). In short, elemental analysis with XPS in Table S1† reveals the nitrogen presence in MOR. The results suggest that NH_4_^+^ has exchanged with NaMOR to form the acidic form of MOR after calcination. Temperature desorption carried out for HMOR demonstrates that at 773 K, NH_4_^+^ could be retained at the catalyst.^85^ For temperature 823 K, the NH_4_^+^ in the form of NH_3_ has not been detected.^85^ For ANF-HMOR in Table S1,† no nitrogen has been observed. NH_3_-TPD is carried out for ANF-NaMOR and ANF-HMOR to demonstrate an increase in acidity in ANF-HMOR, most probably from HMOR. In this case, ANF-NaMOR is used as a control. ANF-NaMOR has 112 μmol g^−1^ NH_3_ desorbed, whereas ANF-HMOR has 216 μmol g^−1^ NH_3_ desorbed. The general survey of the nanofibers revealed only the presence of aluminum and oxygen. Therefore, we can ensure that neither the ANFs nor hybrid fibers contain impurities from the electrospun precursors. Additionally, to verify the MOR content in ANFs, XRD is performed. The XRD patterns of NaMOR and HMOR nanocrystals and the hybrid ANFs are presented in Fig. 2. First, we investigate the crystallographic features of NaMOR before and after ion exchange with NH_4_^+^. The XRD patterns of NaMOR and HMOR are presented in Fig. 2a. The XRD pattern for NaMOR shows diffraction peaks at 2*θ* = 9.8° (200), 13.5° (111), 19.7° (330), 22.3° (150), 25.7° (202), 26.4° (350), 27.7° (511), and 31.0° (402) which match with those for MOR in the MOR (2*θ* = 9.8°, 13.5°, 19.6°, 22.3°, 25.7°, 26.3°, 27.5°, and 30.9°).^86–88^ HMOR presents similar diffraction peaks to NaMOR (2*θ* = 9.8°, 13.5°, 19.7°, 22.4°, 25.7°, 26.4°, 27.7°, and 31°). No other crystallographic phases are observed for both samples. Fig. 2b compares the HMOR nanocrystals with ANF-NaMOR and ANF-HMOR hybrid fibers. In the same figure (Fig. 2b), the XRD pattern of the nanofibers is presented. No crystalline phase has been observed for the ANF sample, confirming the amorphous characteristic of ANFs. ANF-NaMOR and ANF-HMOR diffractograms present peaks that correspond to MOR (Fig. 2a). No other crystallographic phases for the hybrid ANFs are observed.

The effect of MOR has been observed in Fig. 1 with more densified hybrid nanofibers. The presence of MOR in the hybrid fibers became evident with the XRD analysis in Fig. 2. However, an essential aspect is understanding how MOR affects the surface area in the nanofibers. In Table 1, the total surface area of the nanofibers is presented. From the produced catalysts, ANFs possess the highest surface area (192 m^2^ g^−1^), followed by hybrid ANF-HMOR (121 m^2^ g^−1^) and ANF-NaMOR (107 m^2^ g^−1^). The reason for a reduced surface area for nanofibers is that NaMOR or HMOR might block the pore accessibility in ANFs.^54,89,90^

**Table 1:** Fiber diameter, surface area, pore size, and total acidity of ANFs, ANF-NaMOR, ANF-HMOR, Al_2_O_3_-NP, and Al_2_O_3_-HMOR-NP

Sample	Surface area (m^2^ g^−1^)	Total acidity at 373 K (μmol g^−1^)	Total acidity at 373 K (μmol m^−2^)
ANFs	192	178	0.9
ANF-NaMOR	107	116	1.1
ANF-HMOR	121	276	2.3
Al_2_O_3_-NP	162	255	1.6
Al_2_O_3_-HMOR-NP	94	184	2.0

**Fig. 3:**
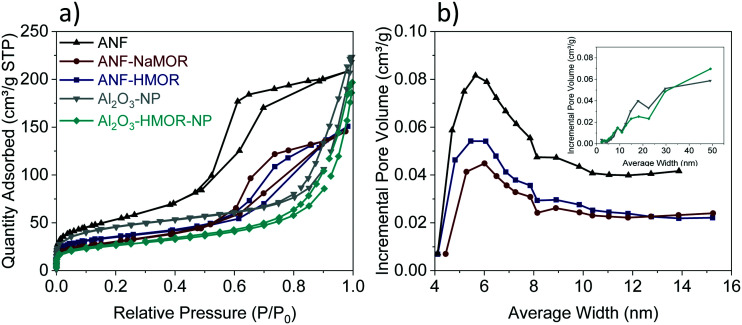
(a) Adsorption–desorption isotherms and (b) pore size distribution for ANFs, ANF-NaMOR, ANF-HMOR, Al_2_O_3_-NP, and Al_2_O_3_-HMOR-NP.

To generate insights into the pore distribution for ANFs with and without MOR, the analysis of the BET isotherms is presented in Fig. 3. For ANFs, ANF-NaMOR, and ANF-HMOR in Fig. 3a, adsorption–desorption isotherms showed hysteresis loops in the multilayer step, which is associated with capillary condensation type IV isotherms for mesopores with H2 hysteresis according to the IUPAC classification.^91^ The hysteresis loop is characteristic of mesoporous materials with cage-like pores or pores with constrictions at the pore opening.^92,93^ The pore distribution plots are presented in Fig. 3b and revealed a wide distribution of pore bodies, with a major pore width distribution around 6 nm for ANFs and hybrid ANFs with NaMOR and HMOR. Fig. 3b shows that ANFs have the highest mesopore and incremental pore volume, followed by ANF-HMOR and ANF-NaMOR. This indicates that MOR modifies the textural properties of ANFs by decreasing the number of pore bodies. The results agree with STEM images in Fig. 1b, d, and f.

Taking the results from the ANFs and hybrid ANFs together, the effect of structuring should be compared with the same type of catalyst but without a nanofiber shape, as shown in Fig. S5.† The Al_2_O_3_-NP and Al_2_O_3_-HMOR-NP control samples have been produced using the same alumina and HMOR precursors. The total surface areas of Al_2_O_3_-NP and Al_2_O_3_-HMOR-NP are presented in Table 1. This table shows a decrease in the surface area of approximately 30 m^2^ g^−1^ for Al_2_O_3_-NP compared to the ANF counterpart. Similar results are observed for Al_2_O_3_-HMOR-NP and the hybrid ANF-HMOR. We hypothesize that the ANFs are less prone to sintering due to restriction of the growth of crystals in the fibers (by confinement) and thus present a higher surface area, as shown in Table 1. When comparing the BET isotherms, variations between Al_2_O_3_-NP or Al_2_O_3_-HMOR-NP are observed. In this case, the type IV adsorption–desorption isotherm shape with an H3 hysteresis loop has been found for Al_2_O_3_-NP and Al_2_O_3_-HMOR-NP (Fig. 3a). Here, the sharp increase at high *P*/*P*_0_ (0.85–0.99) suggests the presence of aggregated slit-shaped pores, which may originate from the interparticle voids. For isotherms with a hysteresis loop at high *P*/*P*_0_, it is likely to observe a wide pore size distribution,^94–97^ as observed in the inset in Fig. 3b. The pore size corresponds to Al_2_O_3_-NP, and Al_2_O_3_-HMOR-NP is 9 nm, similar to the hybrid ANFs. These results might indicate that the hybrid ANFs also provide access for the diffusion of N_2_ molecules, most probably due to the fiber network.^43^

**Fig. 4:**
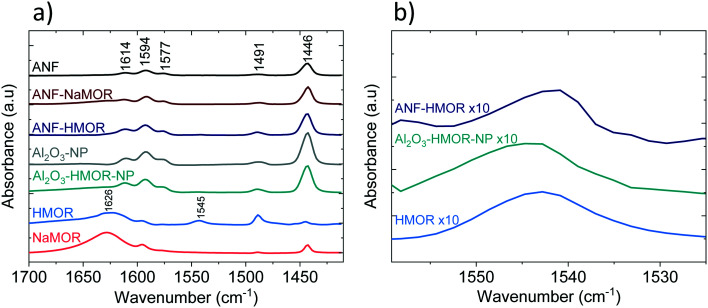
FTIR spectra after Py adsorption at 303 K. a) Full Py range. b) Detailed Py spectra highlighting the BAS band.

Pyridine (Py) is used as a probe molecule to determine the nature of the acid sites (*i.e.*, either LASs or BASs).^98^ In Fig. 4a and b, the FTIR spectra of Py adsorbed at 303 K are presented. The samples composed of alumina (mainly ANFs and Al_2_O_3_-NP), Al_2_O_3_-HMOR-NP, and hybrid alumina (ANF-NaMOR and ANF-HMOR) present an intense band at 1446, 1577, and 1614 cm^−1^. In the case of MOR (NaMOR and HMOR) used for comparison, these peaks are weaker. These bands are attributed to Py adsorbed on LASs, produced by uncoordinated Al^3+^ or cation vacancies.^99^ The signal at 1545 cm^−1^ corresponds to Py adsorbed on BASs (PyH^+^) (Fig. 4b). Among the MOR samples, only HMOR shows an IR band at 1545 cm^−1^. This band confirms that HMOR, ANF-HMOR, and Al_2_O_3_-HMOR-NP contain BASs. The results help to validate the presence of HMOR in the ANFs. NaMOR and ANF-NaMOR do not reveal BASs, and thus, are not tested during DME production. Interestingly, it should be noted that for all samples, two peaks close to 1594 cm^−1^ and 1491 cm^−1^ are present and are assigned to hydrogen-bonded Py and Py adsorbed on both LASs and BASs.^100^

**Fig. 5:**
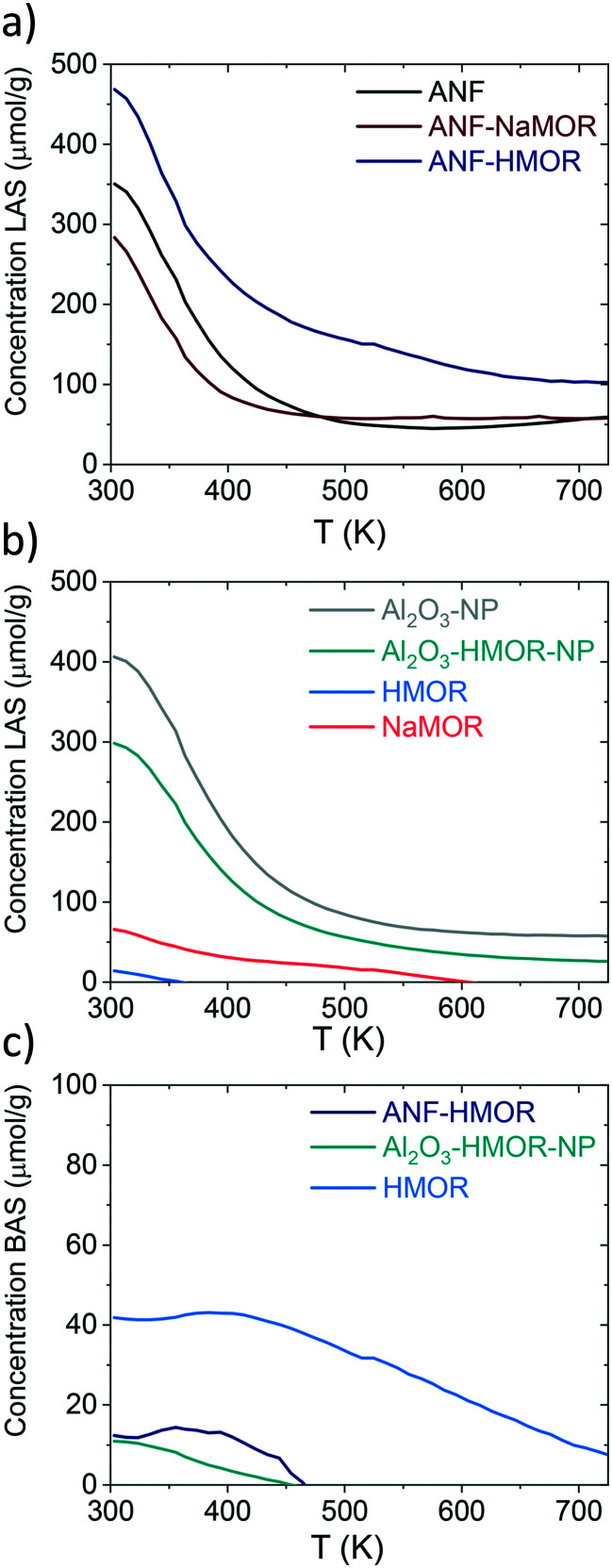
Py-TPD for ANFs, ANF-NaMOR, ANF-HMOR, Al_2_O_3_-NP, Al_2_O_3_-HMOR-NP, HMOR, and NaMOR. In a and b) LASs (μmol g^−1^) and c) BASs (μmol g^−1^) are shown for various samples.

We continued with Py-TPD analysis to determine the amount and the strength of LASs and BASs from Fig. 4. Py-TPD in Fig. 5 shows the LAS and BAS density (expressed in μmol g^−1^) as a function of temperature, estimated using the 1446 cm^−1^ and 1545 cm^−1^ IR bands, where the band at 1446 cm^−1^ is used to estimate the total amount of LASs. Several authors^101–103^ showed that the integrated molar extinction coefficients of LASs do not depend on the nature of oxides, the structure, and the strength of acid sites. Therefore, Emeis's^101^ averaged extinction coefficients of Py adsorption on LASs (2.22 cm μmol^−1^) and BAS (1.67 cm μmol^−1^) are used to quantify the number of sites. For BASs, the IR band at 1545 cm^−1^ is used for quantification.

From Fig. 5a, at 303 K, the hybrid ANF-HMOR has the highest LAS content. This can be correlated to the presence of HMOR. Contrary to ANF-HMOR, ANF-NaMOR shows the lowest LAS concentration, probably because the NaMOR blocks the acid sites of the nanofibers. Lastly, the LAS content remains also low in ANFs. In Fig. 5a, the experiments demonstrate that the LAS content in ANF-HMOR remains higher than that in Al_2_O_3_-NP, Al_2_O_3_-HMOR-NP, or MOR, as shown in Fig. 5b. A possible explanation for ANF-HMOR is that incorporating modifiers on alumina (*e.g.*, HMOR) might promote dealumination, leading to the formation of multiple Al species. Along with HMOR, such Al species can increase the amount of LASs.^104,105^ The results indicate that structured hybrid systems, such as ANF-HMOR, can achieve high acidity, even higher than unstructured acid catalysts (Fig. 5b). The rationale behind our observations is that the nanofiber structure can help densify the acid sites and provide better access to molecules (such as Py), which might be challenging in unstructured acid catalysts (Fig. S5†). Furthermore, Py-TPD reveals that Py desorbs relatively fast from LASs at low temperatures (300–350 K), indicating the presence of weak acid sites (Fig. 5a). At temperatures higher than 350 K, there is a slight decrease in Py desorption, most probably due to the presence of medium and strong acid sites.^43^ Interestingly, after 500 K, all the alumina-modified samples retain similar LASs, except for ANF-HMOR, where Py is still adsorbed at 700 K, suggesting the presence of strongly adsorbed Py species over LASs. The results in Fig. 5a and b (also shown in Table 1) confirm that hybrid ANF-HMOR is the most acidic catalyst (276 μmol g^−1^), followed by ANFs (178 μmol g^−1^) and ANF-NaMOR (116 μmol g^−1^).

In Fig. 5c, HMOR presents the highest BAS density. Py starts desorbing at 400 K and remains adsorbed until 723 K, which means it has the highest Brønsted acid strength. Hybrid ANF-HMOR and Al_2_O_3_-HMOR-NP present lower BAS density due to the lower amount of HMOR. BASs disappear at 460 K in both cases, indicating that sites have a lower BAS strength. It is important to mention that although Py desorption occurs in Fig. 5, the acid sites remain present and can help to catalyze reactions, such as methanol dehydration to DME.

The amount of BASs and LASs for three different temperatures (303, 373, and 423 K) from Fig. 5 is shown in Table 2. In Table 2, ANF-HMOR has the highest acidity and LAS/BAS ratios at 303 K and 373 K, except at 423 K. At 423 K, the BAS content is slightly high for ANF-HMOR (9 μmol g^−1^) and is reflected in Table 2. Overall, Fig. 5 shows that incorporating MOR in alumina modulates the acid site amount and nature.

**Table 2:** Calculated LASs, BASs, total acidity (μmol g^−1^), and LAS/BAS ratios for samples at various selected temperatures

*T*	303 K				373 K				423 K			
Sample	LAS	BAS	Total acidity	LAS/BAS	LAS	BAS	Total acidity	LAS/BAS	LAS	BAS	Total acidity	LAS/BAS
ANFs	351	—	351	—	178	—	178	—	95	—	95	—
ANF-NaMOR	283	—	283	—	117	—	117	—	73	—	73	—
ANF-HMOR	469	12	481	38	276	14	290	20	204	9	213	23
Al_2_O_3_-NP	406	—	406	—	255	—	255	—	148	—	148	—
Al_2_O_3_-HMOR-NP	298	11	309	27	178	6	184	30	101	2	103	49
HMOR	14	42	56	0.33	—	43	43	—	—	42	42	—
NaMOR	66	—	66	—	37	—	37	—	27	—	27	—

### Methanol dehydration to DME

**Fig. 6:**
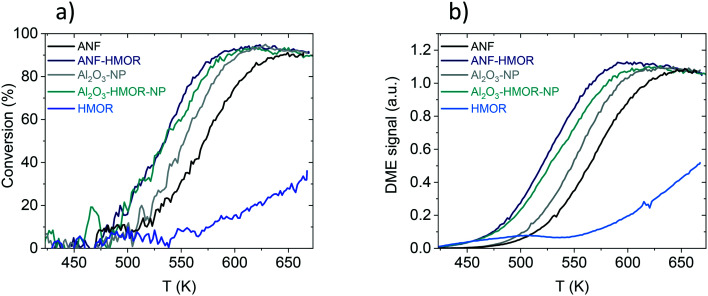
a) Light off curves for methanol conversion. b) Light off curves for DME signal intensity.

The methanol dehydration to DME is assessed as a proof of concept reaction to underline the functionality of the hybrid nanofibers. Fig. 6a shows the methanol conversion of ANFs, ANF-HMOR, Al_2_O_3_-NP, Al_2_O_3_-HMOR-NP, and HMOR over a temperature range of 423 and 673 K. From these catalysts, HMOR starts converting the methanol at lower temperatures (<423 K) compared to Al_2_O_3_-NP and ANFs. However, for temperatures higher than 523 K, the alumina materials are very active and reach the equilibrium conversion at *ca.* 613 K (Al_2_O_3_-NP) and 648 K (ANFs). At 673 K, *ca.* 38% of conversion is reached for HMOR, while for Al_2_O_3_-NP and ANFs, the conversion is *ca.* 90%. Note that the measured conversion corresponds to the equilibrium conversion. The low conversion of HMOR can be related to the low amount of acid sites over the explored temperature range shown in Fig. 5 and 6a.

A comparison between Al_2_O_3_-NP and ANFs is also assessed. Al_2_O_3_-NP is more active than the ANFs, probably due to the higher amount of acid sites per weight of catalysts. Interestingly, the hybrid ANF-HMOR is more active than the Al_2_O_3_-NP and ANFs. This includes the light-off curves of ANF-HMOR and Al_2_O_3_-HMOR-NP, which are very similar. Such similarities include the methanol conversion temperature, which starts at 473 K and reaches the equilibrium conversion at *ca.* 598 K. However, variations in the DME signal intensity are observed in Fig. 6b for ANF-HMOR and Al_2_O_3_-HMOR-NP. In this case, the results indicate that ANF-HMOR is more selective (Table 3) to DME than the other acid catalysts.

**Table 3:** Methanol conversion (%) and DME selectivity

Sample	Methanol conversion (%) at 573^a^ K	DME selectivity (relative intensity) at 573^a^ K	Conversion at *T*_50_^a^ (K)	DME selectivity (relative intensity) at *T*_50_^a^ (K)	Apparent activation energy (kJ mol^−1^)
ANFs	46	1	573	0.9	96 ± 1
ANF-HMOR	87	1	534	1	99 ± 3
Al_2_O_3_-NP	72	0.9	553	0.8	110 ± 1
Al_2_O_3_-HMOR-NP	80	0.9	535	0.8	108 ± 4
HMOR	10	0.9	>673	—	84 ± 4

a The results are derived from the light-off curves, where the experimental error is lower than 15%.

It is then important to compare the conversion (Fig. 6a) and selectivity (Table 3) at 573 K for ANF, ANF-HMOR, Al_2_O_3_-NP, Al_2_O_3_-HMOR-NP, and HMOR. ANF-HMOR remains the highest in Fig. 6a and Table 3, followed by Al_2_O_3_-HMOR-NP, ANFs Al_2_O_3_-HMOR-NP, Al_2_O_3_-NP, and HMOR. The temperature at 50% conversion (*T*_50_) and DME selectivity are also shown in the same table. Again, ANF-HMOR retains the lowest *T*_50_ and the highest conversion at 573 K and DME selectivity, followed by less selective acid catalysts such as Al_2_O_3_-HMOR-NP, ANFs, and Al_2_O_3_-NP. Interestingly, despite their lower conversion (Fig. 6a), the ANFs are more selective to DME than Al_2_O_3_-NP (Table 3), most probably due to the open structure network and the high surface area among the acid catalysts in Table 1. Furthermore, the apparent activation energy calculated from the rate of DME production *vs.* 1/T is shown in Table 3 to compare the catalyst performance further. The activation energy for the ANFs with (99 kJ mol^−1^) and without (96 kJ mol^−1^) HMOR presents slightly lower values than Al_2_O_3_-NP (108 and 110 kJ mol^−1^). Our results show similar values to other catalysts in the literature. This entails conversion, selectivity, and activation energy.^69,81,106^

We then compare the acidity (Fig. 5 and Table 2) with the catalytic performance (Fig. 6 and Table 3) to generate insights into the ANF-HMOR synergy. It is generally accepted that the catalyst composition, surface area, porosity (*i.e.*, pore size and its distribution), and surface acidity affect the performance of the methanol dehydration reaction to DME.^70^ In this reaction, the catalytic activity depends on the surface acidic properties, such as the total number of acidic sites and their strength. Fig. S6† shows the number of acid sites (μmol g^−1^) and the *T*_50_ (K) as a function of the surface area (m^2^ g^−1^). Here, it is observed that the acid sites do not depend on the surface area, and it also does not directly influence the catalyst activity. Previous reports^70,107^ showed that the catalytic activity could be correlated with the number of acidic sites; however, this behavior has not been observed but provides insight into other factors affecting activity.^75^

**Fig. 7:**
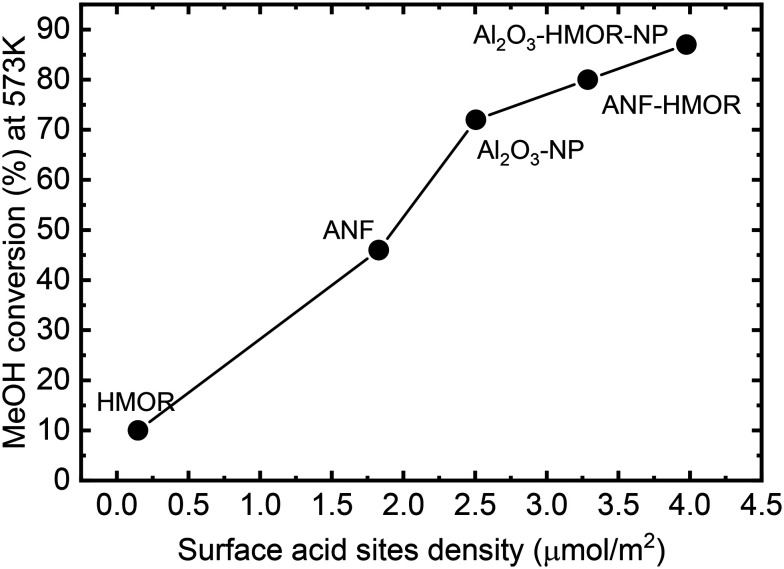
Conversion at 573 K as a function of the acid site density (μmol m^−2^) at 303 K for the unstructured catalysts (HMOR, Al_2_O_3_-NP, and Al_2_O_3_-HMOR-NP) and nanofibers (ANFs and ANF-HMOR).

From the mechanistic point of view, methanol dehydration is considered a bimolecular reaction between two intermediates adsorbed on adjacent surface sites and requires the proximity of two acid sites with adequate acidity.^81^ Thus, increasing the acid site density leads to improved catalyst performance. This becomes evident in Fig. 7, which shows that the conversion increases with the acid site density (μmol m^−2^). The synergy between LASs and BASs produced by interfacial interaction also enhances the methanol dehydration rate.^108^ Therefore, the addition of HMOR in the ANFs and Al_2_O_3_-NP modifies both the acid site density and the acid types by incorporating BASs, thus improving the catalyst performance.

In addition to the nature and strength of the acid sites, the textural properties, such as the surface area, porosity, and average pore diameter, can affect the catalytic performance in dehydration reactions.^69,81^ The BET isotherms (Fig. 3b) suggest the presence of aggregated slit-shaped pores, which may originate from the interparticle voids in Al_2_O_3_-NP and Al_2_O_3_-HMOR-NP. Additionally, they present a wide pore size distribution. In contrast, the pore size distribution plots revealed a wide distribution of pore bodies, with a major pore width distribution around 6 nm for ANFs and hybrid ANFs with NaMOR and HMOR. Furthermore, compared to the NPs that form agglomerates and lack structured macroporosity (Fig. S5†), the nanofibers present a lower diffusion length (*d* = 240–320 nm). This is expected due to the non-woven nanofiber structure that contains a macropore mesh.^43^ These differences in the average pore size, pore size distribution, and diffusion length may explain the variations in selectivity. In Al_2_O_3_-NP and Al_2_O_3_-HMOR-NP, the products could have a higher retention time leading to by-products, such as CO and hydrocarbons, thus decreasing the DME selectivity. Our results suggest that hierarchical structures like fibers and HMOR can enhance the chemical reaction selectivity synergistically.

Based on the catalytic tests used to highlight the hybrid nanofibers' functionality, we can conclude that a higher conversion of methanol to DME is achieved when samples contain high acid site densities and both types of acid sites (LASs and BASs). The presence of both types of acid sites provides synergy effects that positively influence the activity towards the methanol dehydration to DME. Additionally, the fiber morphology favors the DME selectivity. Further studies on ANF-HMOR materials could optimize the amount of BASs and LASs to maximize the conversion and selectivity under dehydration reaction conditions.

## Conclusions

Hybrid ANFs with high acidity have been synthesized using electrospinning. Acid site tunability is possible in these nanofibers using MOR nanocrystals. The nanofibers have shown multilevel pore combinations, such as mesopores and macropores. IR-Py demonstrates the nature type and desorption strength of the acid sites in ANF-HMOR, which prevail between 423 and 673 K. The methanol dehydration reactions showed the advantage of ANF-HMOR synergistically contributing to the increase in methanol conversion and DME selectivity.

## Conflicts of interest

There are no conflicts to declare.

## Supplementary Material

CY-012-D2CY00143H-s001
