# Supplementary material for: Tuning the catalytic acidity in Al2O3 nanofibers with mordenite nanocrystals for dehydration reactions
Source: Catal Sci Technol. 2022 May 11;12(13):4243–54. doi: 10.1039/d2cy00143h (PMC9252259; doi:10.1039/d2cy00143h)
Supplement: CY-012-D2CY00143H-s001 [file CY-012-D2CY00143H-s001.pdf]

Electronic Supplementary Material (ESI) for Catalysis Science & Technology.
This journal is © The Royal Society of Chemistry 2022

Electronic supporting information (ESI)

Tunning catalytic acidity in Al_2O_3 nanofibers with mordenite nanocrystals for dehydration reactions

M.A. Rodriguez-Olguin[1,#], R. N. Cruz-Herbert[2,#], H. Atia[3], M. Bosco[4,5], E.L. Fornero[4,6], R. Eckelt[3], D. A. De Haro Del Río[2,], A. Aguirre[4,*], J.G.E. Gardeniers[1,*], A. Susarrey-Arce[1,*]*

[1]Mesoscale Chemical Systems, MESA+ Institute, University of Twente, PO. Box 217, 7500AE, Enschede, The Netherlands

[2]Universidad Autónoma de Nuevo León, Facultad de Ciencias Químicas, Pedro de Alba S/N., San Nicolás de los Garza, Nuevo León, 64455, México

[3]Leibniz Institute for Catalysis, Albert-Einstein-Straße 29a, D-18059, Rostock, Germany

[4]Instituto de Desarrollo Tecnológico para la Industria Química (INTEC), Universidad Nacional del Litoral, CONICET, Güemes 3450, S3000GLN, Santa Fe, Argentina

[5]Facultad de Ingeniería Química, Universidad Nacional del Litoral (UNL), Santiago del Estero 2829, Santa Fe, 3000, Argentina

[6]Facultad de Ingeniería en Ciencias Hídricas, UNL, Ciudad Universitaria. Ruta Nacional N° 168 - Km 472,4, 3000 Santa Fe, Argentina.

[#]These authors contributed equally to this work

Corresponding authors: A. Aguirre (aaguirre@santafe-conicet.gov.ar); D. A. De Haro Del Río (david.dharodlr@uanl.edu.mx); J.G.E. Gardeniers (j.g.e.gardeniers@utwente.nl); A. Susarrey-Arce (a.susarreyarce@utwente.nl)

Electronic supporting information (ESI)

Table of content

Content	Page number
**Figure S1**. a) SEM images of commercial NaMOR with crystallite size of 222 nm estimated with XRD and b) Electrospun hybrid ANFs containing HMOR after annealing.	3
**Figure S2**. STEM image of a) HMOR and b) NaMOR zeolite seeds. The particle sizes of NaMOR and HMOR correspond to 118nm and 110nm as estimated with XRD.	4
**Figure S3**. EDS mapping of ANF-HMOR. Silicon signal is color-coded in blue, Oxygen signal in red, and Al signal in green.	5
**Figure S4**. Chemical composition at the surface of a) NaMOR, b) HMOR, c) ANFs, d) ANF-NaMOR, and e) ANF-HMOR obtained with XPS.	6
**Table S1**. Atomic percentage (at.%) of the different samples evaluated with XPS in **Figure S4**.	7
**Figure S5**. SEM images of a) Al_2O_3-NP and b) Al_2O_3-HMOR-NP.	8
**Figure S6**. Acid sites (μmol/g) and T_{50} (K) as a function of the surface area (m^2/g) for the unstructured catalysts (HMOR, Al_2O_3-NP, and Al_2O_3-HMOR-NP) and nanofibers (ANF, and ANF-HMOR).	9

Electronic supporting information (ESI)

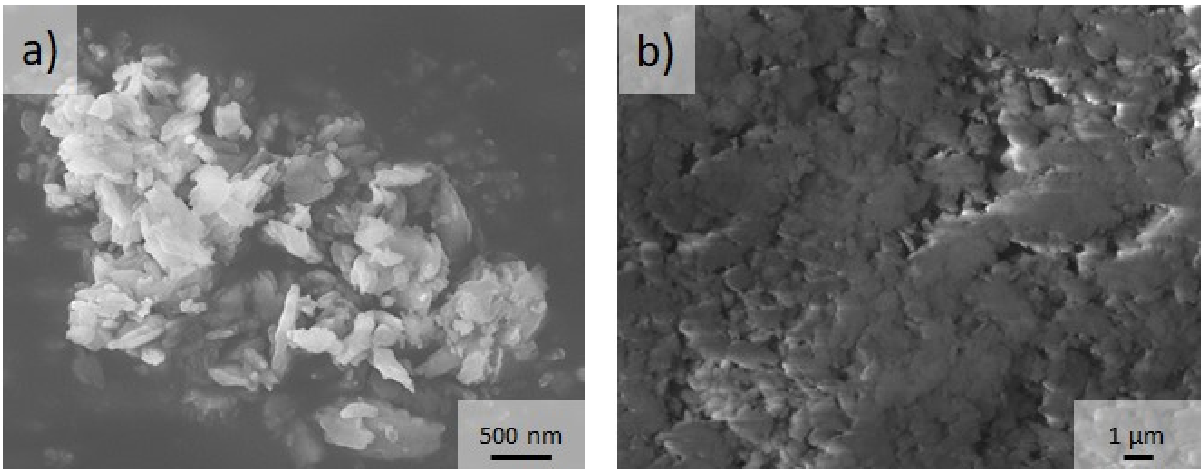

Figure S1. a) SEM images of commercial NaMOR with crystallite size of 220 nm estimated with XRD and b) Electrospun hybrid ANFs containing HMOR after annealing.

Electronic supporting information (ESI)

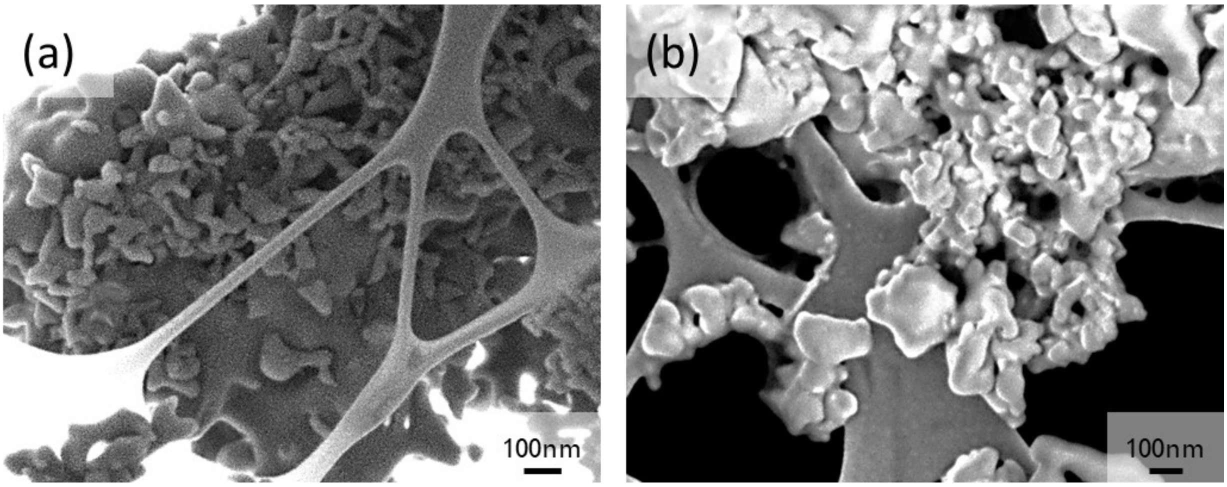

Figure S2. STEM image of a) HMOR and b) NaMOR zeolite seeds. The particle sizes of NaMOR and HMOR correspond to 118 nm and 110 nm as estimated with XRD.

Electronic supporting information (ESI)

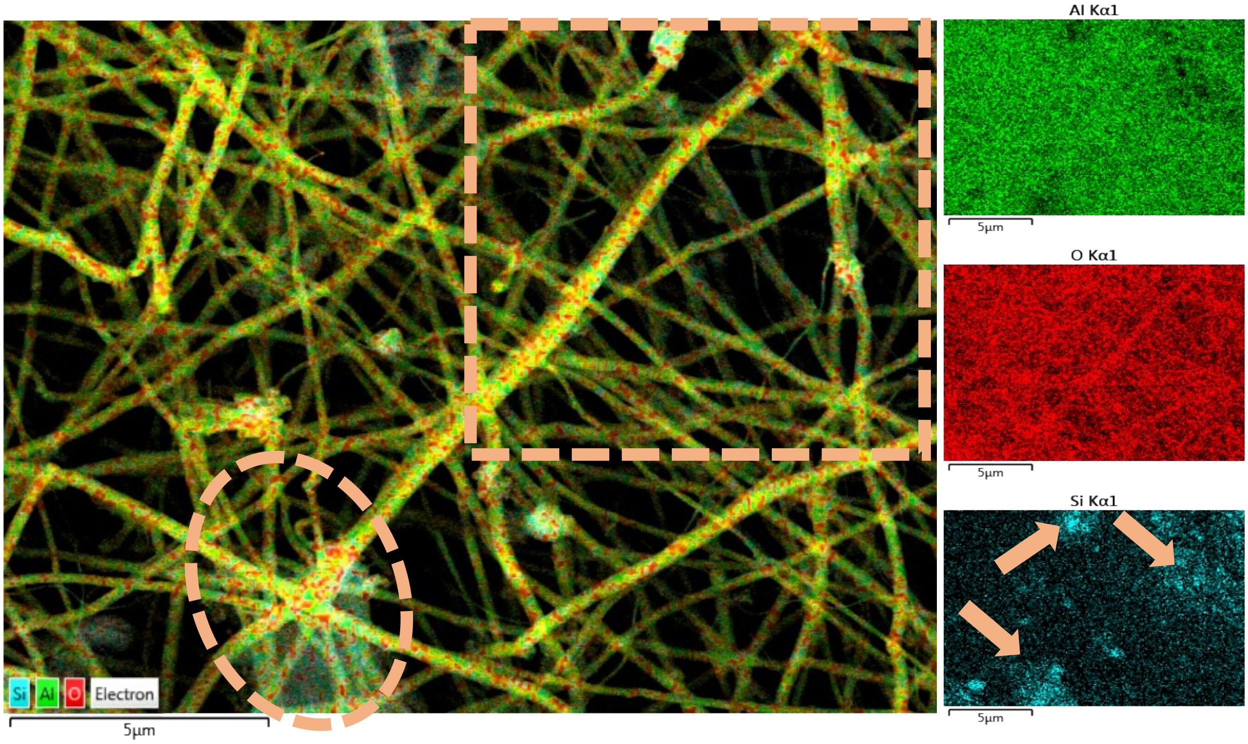

Figure S3. EDS mapping of ANF-HMOR. Silicon signal is color-coded in blue, Oxygen signal in red, and Al signal in green.

The presence of HMOR in the ANFs is indirectly confirmed with EDS. HMOR composition is mainly aluminum (green), oxygen (red), and silicon (blue), while ANFs are composed of aluminum and oxygen. Aluminum and oxygen signals have a color overlap, leading to yellow color. However, EDS shows the presence of silicon, which can be related to MOR nanocrystals. MOR has been found denser over the nanofiber architectures (see the dashed square in light orange). MOR has also been found distributed out of the non-woven nanofiber network as inhomogeneity (see the dashed ellipse in light orange). To facilitate the reader, compare with the EDS signal of silicon (right bottom panel). Here, denser silicon signals are highlighted with light orange arrows.

Electronic supporting information (ESI)

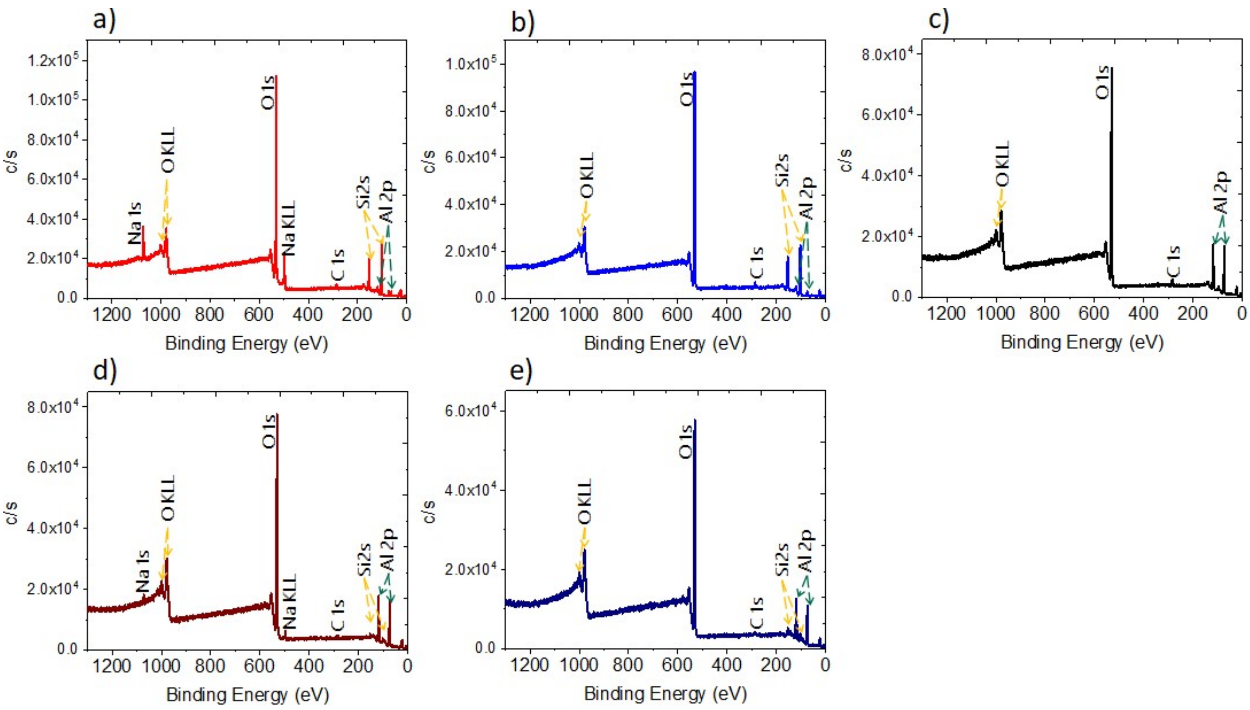

Figure S4. Chemical composition at the surface of a) NaMOR, b) HMOR, c) ANFs, d) ANF-NaMOR, and e) ANF-HMOR obtained with XPS.

Electronic supporting information (ESI)

Table S1. Atomic percentage (at.%) of the different samples evaluated with XPS in **Figure S4**.

Sample	C	Si/Al	O	Na	N
NaMOR	3.6	7.9	64.1	6.53	0.1
HMOR	4.9	7.3	64.5	0.3	2.3
ANFs	5.2	26.9 at.% of Al	67.7	-	-
ANF-NaMOR	2.5	0.05	68.5	1.6	-
ANF-HMOR	2.5	0.10	68.7	-	-

Table S1 shows the presence of silicon, aluminum, oxygen, and sodium in NaMOR. HMOR contains the same elements plus nitrogen, indicating the presence of NH_4^+. The ANF-NaMOR and ANF-HMOR show the same elemental composition. A decrease in the Si/Al has been seen compared to NaMOR or HMOR. Furthermore, no nitrogen has been observed for ANF-HMOR. However, the detection of Na and Si indicates the presence of MOR in the hybrid ANFs. It should be noted that the low Si/Al is expected due to the high Al content of the ANFs. The general XPS survey in Table S1 revealed the presence of C 1s in all cases.

Electronic supporting information (ESI)

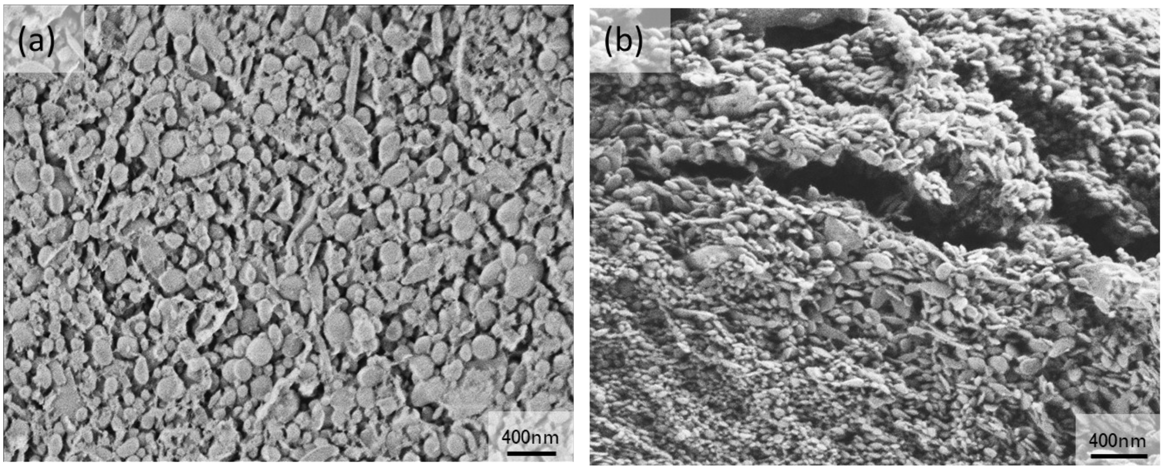

Figure S5. SEM images of a) Al_2O_3-NP and b) Al_2O_3-HMOR-NP.

Electronic supporting information (ESI)

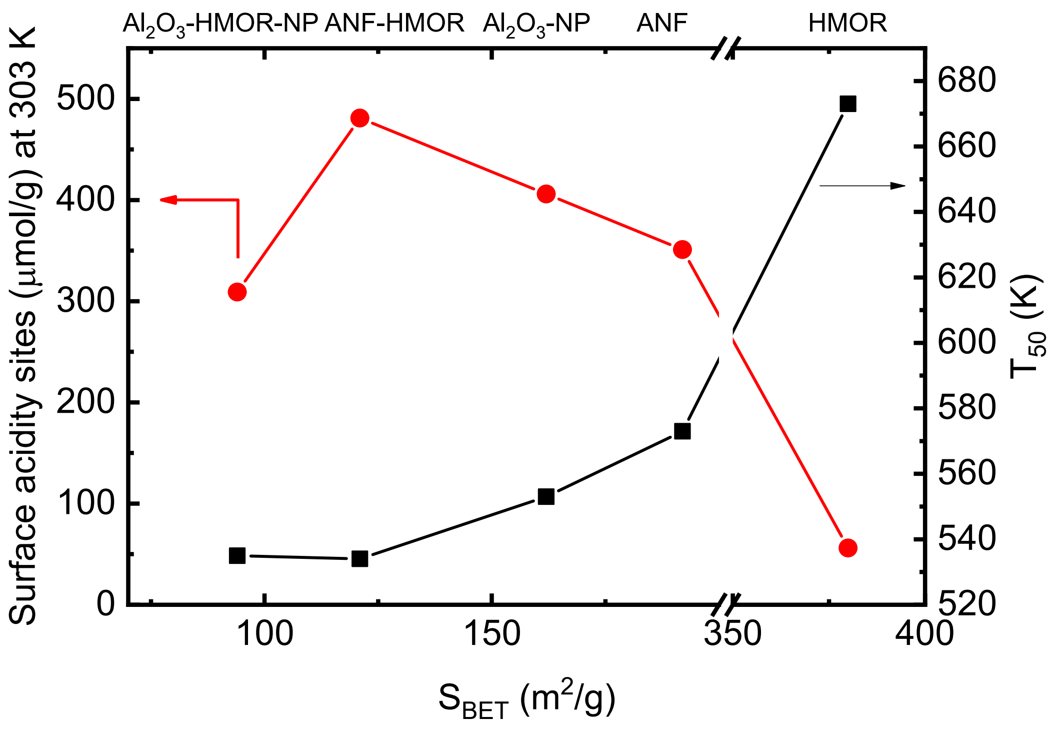

Figure S6. Acid sites (µmol/g) and T_{50} (K) as a function of the surface area (m^2/g) for the unstructured catalysts (HMOR, Al_2O_3-NP, and Al_2O_3-HMOR-NP) and nanofibers (ANF, and ANF-HMOR).
